# Community differentiation and assembly of soil *nosZ*-type denitrifiers across dominant plant communities in an alpine wetland of Qinghai Lake

**DOI:** 10.3389/fmicb.2026.1972695

**Published:** 2026-10-23

**Authors:** Shijia Zhou, Ni Zhang, Jiao Tang, Kelong Chen

**Affiliations:** 1 Qinghai Province Key Laboratory of Physical Geography and Environmental Process, College of Geographical Science, Qinghai Normal University, Xining, China; 2 Key Laboratory of Tibetan Plateau Land Surface Processes and Ecological Conservation (Ministry of Education), Qinghai Normal University, Xining, China; 3 National Positioning Observation and Research Station of Qinghai Lake Wetland Ecosystem in Qinghai, National Forestry and Grassland Administration, Haibei, Qinghai, China

**Keywords:** alpine wetland, community assembly, dominant plant communities, nitrous oxide reduction, *nosZ*, Qinghai Lake

## Abstract

**Background:**

Nitrous oxide (N₂O) reduction mediated by *nosZ*-harboring microorganisms represents the terminal step of denitrification and an important biological sink for N₂O in wetland soils, yet how naturally occurring dominant plant communities are associated with the diversity, taxonomic differentiation, and assembly of *nosZ*-type communities in alpine wetlands remains poorly understood.

**Methods:**

We investigated four dominant plant communities in wetlands surrounding Qinghai Lake on the northeastern Qinghai–Tibet Plateau, dominated by *Blysmus sinocompressus* (BS), *Carex regescens* (CR), *Elymus nutans* (EN), and *Leymus secalinus* (LS), and integrated *nosZ*-targeted amplicon sequencing with vegetation biomass, soil physicochemical properties, environmental-association analyses, and phylogeny-based null models to evaluate variation in community diversity, taxonomic composition, environmental correlates, and assembly processes.

**Results:**

The four plant communities exhibited pronounced differences in vegetation–soil conditions and supported distinct *nosZ*-type communities. LS-associated soils showed the highest richness and the largest pool of community-specific OTUs, whereas Proteobacteria and *Bradyrhizobium* dominated the overall taxonomic composition. Environmental associations differed between community diversity and taxonomic structure: diversity was more strongly associated with plant biomass, soil temperature, and carbon and nitrogen status, whereas compositional variation was more closely related to soil C ratio, pH, total carbon, moisture, and nitrate nitrogen. Phylogeny-based null-model analysis further indicated that community assembly was predominantly associated with variable selection, although the relative contributions of dispersal limitation and other stochastic processes differed among dominant plant communities.

**Conclusion:**

These findings demonstrate that dominant plant communities in the Qinghai Lake alpine wetland are associated with coordinated differentiation in vegetation–soil environments, *nosZ*-type community composition, and assembly processes, thereby characterizing the taxonomic differentiation and inferred assembly of the primer-defined soil *nosZ* community across contrasting plant-community habitats; however, the implications for realized N₂O reduction require direct validation using gene-abundance, transcriptional, enzyme-activity, and gas-flux measurements.

## 1 Introduction

Alpine wetlands are biogeochemically active ecosystems in which strong hydrological and redox gradients support tightly coupled microbial nitrogen transformations. These processes are particularly relevant to nitrous oxide (N₂O), a long-lived greenhouse gas whose increasing atmospheric burden contributes to both climate forcing and stratospheric ozone depletion (Ravishankara et al., 2009; Tian et al., 2020). During denitrification, nitrate is sequentially reduced through nitrite, nitric oxide, and N₂O to dinitrogen, such that N₂O can either accumulate as an intermediate or be further consumed. The terminal reduction of N₂O to N₂ is catalyzed by nitrous oxide reductase, encoded by the *nosZ* gene, and represents the only known enzymatic biological sink for N₂O (Sanford et al., 2012; Jones et al., 2013). Importantly, *nosZ* is distributed not only among canonical denitrifiers but also among phylogenetically diverse microorganisms lacking a complete denitrification pathway, substantially broadening the recognized diversity of N₂O-reducing communities; experimental evidence further demonstrates that such non-denitrifying N₂O reducers can measurably decrease soil N₂O production (Sanford et al., 2012; Domeignoz-Horta et al., 2016). This functional guild may be particularly important in environmentally heterogeneous alpine wetlands, where variation in hydrological and edaphic conditions can reorganize *nosZ*-type denitrifier communities. Indeed, recent work in the source wetlands of Qinghai Lake showed that experimentally altered precipitation changed soil conditions and was accompanied by shifts in *nosZ*-type community composition and assembly processes (Zhang et al., 2025). Understanding the diversity and organization of *nosZ*-harboring microorganisms is therefore important for elucidating how alpine wetland environments are associated with the microbial balance between N₂O production and consumption.

Vegetation can influence denitrifier communities indirectly by modifying the physicochemical and resource environment in which these microorganisms occur. In wetlands, differences among dominant plant species in root biomass, rhizodeposition, litter inputs, and rhizosphere conditions can alter the availability of labile carbon and nitrogen substrates, soil moisture, and other edaphic properties that constrain denitrification and denitrifier distributions (Le Roux et al., 2013; Wu et al., 2017; Fang et al., 2023). At the ecosystem level, a meta-analysis of 419 estimates showed that wetland vegetation enhanced denitrification on average, but the magnitude of this effect differed among communities dominated by different plant species, highlighting the importance of plant identity and associated habitat conditions for microbial N removal (Alldred and Baines, 2016). Such vegetation-associated differentiation is also evident at the microbial community level. Natural meadow and forest soils harbored distinct *nosZ*-type denitrifier assemblages, with community composition associated with vegetation type and soil C:N conditions (Rich et al., 2003), while rhizospheres of different wetland plant species supported differences in *nosZ* community structure and genotype diversification (Ruiz-Rueda et al., 2009). Root-derived carbon provides one plausible pathway linking vegetation to these patterns: root exudate composition and release vary among wetland plant species and have been associated with changes in denitrifier abundance, richness, and community structure (Wu et al., 2017), whereas controlled microcosm experiments have shown that the responses of *nosZ*-I communities to carbon supply depend strongly on soil water-filled pore space (Langarica-Fuentes et al., 2018). Soil pH and moisture can likewise alter denitrifier abundance and composition, including *nosZ*-I-bearing communities, further indicating that vegetation effects are likely mediated through interacting biotic and edaphic filters rather than plant identity alone (Bowen et al., 2020). Collectively, these findings suggest that dominant plant communities can be associated with distinct belowground environmental templates for *nosZ*-harboring microorganisms; however, how such plant-associated environmental heterogeneity translates into differences in the organization and assembly of *nosZ*-type denitrifier communities in natural alpine wetlands remains poorly resolved.

Although differences in microbial diversity and community composition can reveal how *nosZ*-harboring microorganisms vary among habitats, these patterns alone do not identify the ecological processes responsible for community formation. Microbial community assembly is jointly influenced by deterministic processes, such as environmental selection and biotic interactions, and stochastic processes, including dispersal, dispersal limitation, and ecological drift, with their relative importance varying across environmental contexts (Stegen et al., 2012; Stegen et al., 2013). Phylogeny-based null-model frameworks provide a means of moving beyond descriptive community comparisons by partitioning compositional turnover into processes associated with variable or homogeneous selection and, when selection is weak, with dispersal-related or undominated stochastic processes (Stegen et al., 2013). Emerging evidence indicates that such assembly processes are also relevant to N₂O-reducing microbial guilds. Root-associated *nosZ* communities can be structured by interactions between plant identity and soil type, demonstrating that the establishment of N₂O-reducing communities depends on both plant-associated and edaphic filters (Graf et al., 2022). Moreover, the balance between deterministic and stochastic assembly of denitrifier communities is not fixed: long-term tillage altered the relative contribution of these processes in *nosZ*-bearing communities, while irrigation shifted *nosZ*-type denitrifier assembly from predominantly stochastic toward deterministic organization (Wang et al., 2022; Yu et al., 2023). Particularly relevant to alpine wetlands, a precipitation-manipulation experiment in the source wetland of Qinghai Lake found that *nosZ*-type community assembly was dominated by deterministic processes and changed alongside precipitation-induced shifts in soil conditions (Zhang et al., 2025). However, current evidence has largely arisen from agricultural management experiments, controlled plant–soil systems, or manipulation of individual environmental factors. Whether naturally occurring dominant plant communities in alpine wetlands are associated with distinct *nosZ* community assembly regimes, and whether observed compositional differentiation primarily reflects heterogeneous environmental selection or stochastic processes such as dispersal limitation and drift, therefore remains comparatively poorly resolved. Integrating taxonomic diversity, community composition, environmental associations, and null-model-based assembly analyses is thus necessary to clarify the ecological processes underlying the spatial organization of *nosZ*-type denitrifiers in heterogeneous alpine wetland habitats.

In this study, we characterized soil *nosZ*-type denitrifier communities associated with four naturally occurring plant-community habitats around Qinghai Lake. We asked whether soil *nosZ* diversity and composition differed among these habitats, which measured vegetation and soil variables covaried with the observed community patterns, and whether null-model-inferred assembly processes differed among habitats. Because plant-community type and site conditions covaried in this observational design, the analyses were intended to identify habitat associations rather than causal effects of plant identity.

## 2 Materials and methods

### 2.1 Study area and sampling

The study was conducted in wetlands surrounding Qinghai Lake on the northeastern Qinghai–Tibet Plateau, China. The Qinghai Lake Basin extends from 36°15′ to 38°20′N and from 97°50′ to 101°20′E, covering an area of approximately 29,661 km^2^. The region lies at the intersection of the eastern monsoon, northwestern arid, and southwestern alpine climatic zones of China and is characterized by a semiarid alpine climate. The mean elevation exceeds 3,000 m above sea level, the mean annual temperature ranges from −4.0 to 4.6 °C, and annual precipitation ranges from 291.0 to 579.0 mm, with most rainfall occurring between June and September. Vegetation and soil types are spatially heterogeneous across the basin, with *Blysmus sinocompressus*, *Leymus secalinus*, *Elymus nutans*, and *Carex regescens* among the dominant wetland plant species.

Soil sampling was conducted in August 2020 during the peak growing season at 10 spatially separated sampling sites distributed around Qinghai Lake. The locations of all sampling sites and their corresponding dominant plant-community types are shown in Supplementary Figure S1. Each site was assigned to one of four dominant plant-community types according to the prevailing vegetation: *Blysmus sinocompressus* (BS), *Carex regescens* (CR), *Elymus nutans* (EN), or *Leymus secalinus* (LS). The sampling design comprised two BS sites (10 quadrats), two CR sites (10 quadrats), three EN sites (15 quadrats), and three LS sites (15 quadrats), giving a total of 10 sites and 50 quadrats. At each site, five spatially separated 1 × 1 m quadrats with relatively flat topography and homogeneous vegetation were established. Within each quadrat, five subsampling points were positioned along an S-shaped transect, and surface soil was collected from 0 to 10 cm depth using a 4.5-cm-diameter soil auger. The five subsamples collected within each quadrat were thoroughly homogenized to form one composite soil sample, resulting in 50 independently collected and sequenced quadrat-level soil samples. Visible roots and plant residues were removed, and the soil was passed through a 2-mm sieve before subsequent physicochemical and molecular analyses. The quadrat represented the unit of field sampling and laboratory measurement, whereas the sampling site constituted the higher-level spatial unit and the independent spatial replicate for inference concerning plant-community type. Because the five quadrats within each site shared the same site-level environmental context, they were treated as subsamples nested within sites rather than as five fully independent spatial replicates in inferential analyses. The present study was based on the same field sampling campaign and sampling framework as our previously published investigation of *nirK*-type denitrifier communities in the Qinghai Lake wetlands (Tang et al., 2026). The vegetation biomass and soil physicochemical data were collected during this shared sampling campaign and have been reported previously. In contrast to the previous investigation, which focused on *nirK*-harboring communities, the present study specifically examined the diversity, taxonomic differentiation, environmental associations, and inferred assembly processes of *nosZ*-type denitrifier communities.

### 2.2 Vegetation biomass and soil physicochemical measurements

Soil ammonium nitrogen (NH₄^+^-N) and nitrate nitrogen (NO₃^−^-N) were determined using the sodium salicylate method and hydrazine sulfate reduction method, respectively, with a continuous-flow analyzer (FUTURA, France). Total carbon (TC) and total nitrogen (TN) were measured using an elemental analyzer (Vario EL III, Elementar Analysensysteme GmbH, Germany). Soil pH was determined in a 1:2.5 soil-to-water suspension using a pH meter (Mettler Toledo, Switzerland). Soil temperature was measured *in situ* using a soil thermometer, and soil moisture was determined using a soil moisture meter.

Aboveground biomass (AGB) was determined by clipping living aboveground vegetation from one-quarter of each quadrat. Belowground biomass (BGB) was collected using the soil-core method. Soil cores from the five subsampling points within each quadrat were combined and sieved, after which roots were separated from the soil, washed, and living roots were retained based on tissue elasticity and color. Above- and belowground biomass samples were dried at 60 °C to constant mass before weighing.

### 2.3 DNA extraction, *nosZ* amplification, sequencing, and sequence processing

Total soil DNA was extracted from 0.4 to 0.5 g of soil using the PowerSoil DNA Isolation Kit (Mo Bio Laboratories, USA), and DNA quality and integrity were assessed by electrophoresis on a 2% agarose gel at 80 V for 40 min. The *nosZ* gene was amplified using the primers *nosZ*-F (5′-CGYTGTTCMTCGACAGCCAG-3′) and 1622R (5′-CGSACCTTSTTGCCSTYGCG-3′). PCR amplification was performed in a 20-μL reaction using an ABI GeneAmp 9700 thermocycler under the following conditions: initial denaturation at 95 °C for 5 min; 30 cycles of denaturation at 95 °C for 30 s, annealing at 55 °C for 30 s, and extension at 72 °C for 45 s; followed by a final extension at 72 °C for 10 min. PCR products were purified using an AxyPrep PCR purification kit (Axygen, USA) and sequenced on an Illumina HiSeq 2500 platform.

Raw paired-end sequences were merged using PANDAseq, quality filtered using PRINSEQ, and screened for chimeric sequences using USEARCH. Clean sequences were clustered into operational taxonomic units (OTUs) at 97% sequence similarity using UCLUST. Subsequent community analyses were conducted using the resulting OTU abundance and taxonomic annotation tables.

### 2.4 Community diversity and taxonomic composition

Sequencing depth was evaluated using rarefaction curves. Alpha diversity of *nosZ*-type denitrifier communities was characterized using Chao1, ACE, Shannon diversity, and Simpson diversity indices. These metrics were originally calculated in QIIME 1.9.1 and R 4.0.5 using the MicrobiotaProcess and phyloseq packages. Community compositional differences among BS, CR, EN, and LS were visualized by non-metric multidimensional scaling (NMDS) based on community dissimilarity, and statistical differences among plant-community types were evaluated using permutational multivariate analysis of variance (PERMANOVA). Homogeneity of multivariate dispersion was additionally assessed using PERMDISP before interpreting PERMANOVA results.

Shared and plant-community-specific OTUs were summarized using intersection analyses. Taxonomic composition was characterized at the phylum and genus levels, with major taxa identified according to their relative abundances. Community compositional patterns were visualized using principal component analysis (PCA) and non-metric multidimensional scaling (NMDS). Differences in community composition among plant-community habitats were evaluated using PERMANOVA and ANOSIM.

### 2.5 Environmental associations and community assembly

Associations between *nosZ*-type denitrifier communities and vegetation–soil environmental variables were evaluated using complementary multivariate approaches. Relationships between dominant genera and individual environmental variables were examined using correlation analysis. Mantel tests were further used to assess associations between community dissimilarity matrices and environmental variables. Associations between *nosZ*-type denitrifier communities and vegetation–soil environmental variables were evaluated using correlation and Mantel analyses. Prior to environmental association analyses, correlations among environmental variables were examined to identify potential collinearity. Mantel tests were used to evaluate associations between community dissimilarity and individual vegetation–soil variables, with statistical significance assessed by permutation. Mantel tests were conducted at both the phylum and genus levels to evaluate whether environmental associations were consistent across different taxonomic resolutions. The phylum level was used to characterize broad lineage-level responses, whereas the genus level provided a finer and more ecologically interpretable representation of taxonomic compositional variation. Because the complete OTU matrix was highly dimensional and sparse, OTU-level data were used primarily for analyses of overall community differentiation, while the phylum- and genus-level matrices were used for taxonomic-scale environmental association analyses.

To infer the ecological processes governing *nosZ*-harboring denitrifier community assembly, the *β*-nearest taxon index (*β*NTI) was calculated based on *β*-mean nearest taxon distance (*β* MNTD) and null distributions generated by randomizations. Pairwise comparisons were categorized according to standard ecological thresholds: |*β*NTI| > 2 indicated deterministic selection (*β*NTI > + 2 for variable selection and *β*NTI <−2 for homogeneous selection). For pairwise comparisons falling within the neutral zone (|*β*NTI| ≤ 2), Raup-Crick Bray-Curtis (RCbray) metrics were further calculated to partition stochastic processes, distinguishing dispersal limitation (RC_bray_ > +0.95), homogenizing dispersal (RC_bray_ < −0.95), and undominated drift (|RC_bray_| ≤ 0.95).

### 2.6 Statistical analyses

All statistical analyses and visualizations were performed primarily in R. Differences in environmental variables and alpha-diversity indices among the four plant-community types were evaluated using nonparametric tests when distributional assumptions were not met, followed by appropriate pairwise comparisons. Community compositional differences were assessed using PERMANOVA, and PERMDISP was used to evaluate homogeneity of within-group multivariate dispersion. Correlations among environmental variables and between environmental properties and microbial attributes were evaluated using Pearson’s or Spearman’s correlation as appropriate, whereas Mantel tests were used for matrix-level associations. Statistical significance was assessed at *p* < 0.05. Where multiple hypotheses were tested simultaneously, *p-*values were adjusted for false discovery rate (FDR) using the Benjamini–Hochberg procedure. Because the sampling design comprised 10 spatially distinct sites with five quadrats nested within each site, the hierarchical sampling structure was retained in inferential analyses. The site was treated as the independent spatial sampling unit. For univariate environmental and alpha-diversity analyses, quadrat-level observations were averaged within each site before comparisons among dominant plant-community types.

## 3 Results

### 3.1 Vegetation and soil properties differed among dominant plant communities

Vegetation and soil properties differed markedly among the four dominant plant communities (Supplementary Figure S2). Aboveground biomass (AGB) was highest in CR, whereas EN and LS showed lower values, with BS exhibiting an intermediate level. Differences in belowground biomass (BGB) were more pronounced, with CR showing the highest BB, followed by BS, whereas EN and LS had substantially lower values. Soil CNR also differed among plant communities, with higher values in BS and CR than in EN and LS. Soil pH showed a similar pattern, with CR having the highest value and EN and LS having comparatively lower values. Total carbon (TC) and total nitrogen (TN) were both lowest in LS, whereas BS, CR, and EN did not differ significantly from one another. Ammonium nitrogen (AN) also varied among plant communities, with LS showing a lower value than CR and EN. Collectively, these differences demonstrated pronounced vegetation–soil environmental heterogeneity among the four dominant plant communities.

### 3.2 Diversity and OTU distribution of *nosZ*-type denitrifiers

Rarefaction curves based on ACE and Chao1 gradually approached saturation with increasing sequencing depth, indicating that the sequencing effort adequately captured the richness patterns of the *nosZ*-type denitrifier communities, When the sequencing depth reached approximately 28,000 reads, the rarefaction curves for all samples plateaued markedly(Figure 1a). The LS samples generally exhibited higher richness estimates than the other plant-community types, whereas BS, CR, and EN showed comparatively lower values.

**Figure 1:**
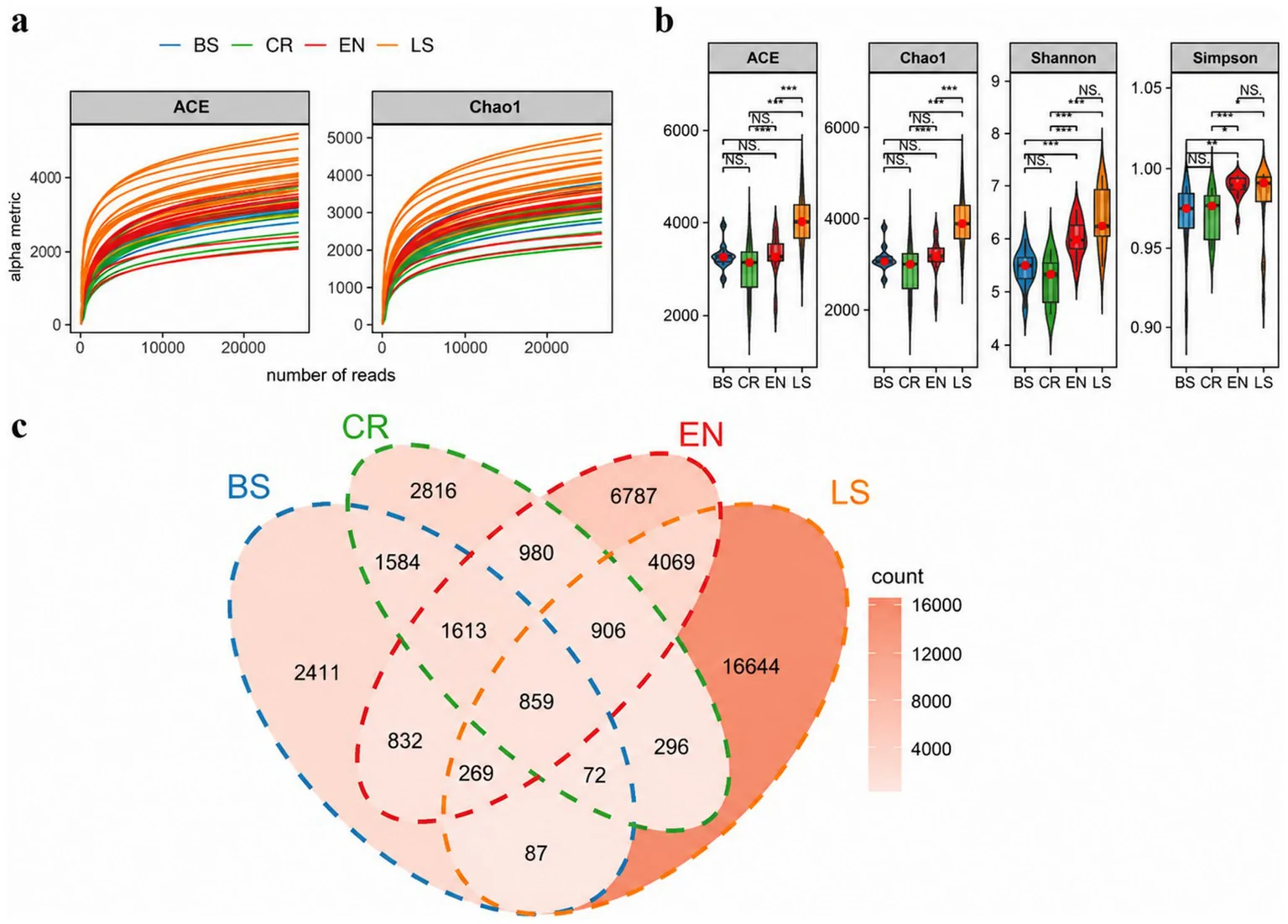
Sequencing coverage, alpha diversity, and OTU distribution of *nosZ*-type denitrifier communities across dominant plant communities. **(a)** Rarefaction curves based on ACE and Chao1 richness estimators for *nosZ*-type denitrifier communities across the four dominant plant-community types. Curves approaching a plateau indicate that sequencing depth was sufficient to capture the major richness patterns. **(b)** Alpha-diversity patterns based on ACE, Chao1, Shannon, and Simpson indices. Violin plots show the distribution of each diversity metric among BS, CR, EN, and LS. Pairwise significance is indicated above the plots, with NS denoting no significant difference and asterisks indicating significant differences. **(c)** Venn diagram showing shared and unique *nosZ* OTUs among the four dominant plant communities. A total of 859 OTUs were shared by all four groups, whereas BS, CR, EN, and LS contained 2,411, 2,816, 6,787, and 16,644 unique OTUs, respectively. BS, *Blysmus sinocompressus*; CR, *Carex regescens*; EN, *Elymus nutans*; LS, *Leymus secalinus*. Asterisks indicate statistical significance (ns indicates no significant difference, *p < 0.05, **p < 0.01, ***p < 0.001, and ****p < 0.0001).

Alpha diversity differed substantially among the four dominant plant communities (Figure 1b). Both ACE and Chao1 richness were highest in LS and lowest in CR, with LS showing significantly higher (*p* < 0.05)values than the other three plant-community types. Shannon diversity was comparatively higher in EN and LS than in BS and CR, whereas EN and LS did not differ markedly from each other. Simpson diversity showed a relatively narrower range of variation, although significant differences were still detected among several plant-community pairs. Overall, the differences among plant communities involved both species richness and diversity components of the *nosZ*-type community.

A total of 40,225 *nosZ* OTUs were recovered after sequence filtering and rarefaction. Only 859 OTUs were shared among all four dominant plant communities, whereas the numbers of community-specific OTUs differed substantially (Figure 1c). BS, CR, EN, and LS contained 2,411, 2,816, 6,787, and 16,644 unique OTUs, respectively. Thus, LS contained by far the largest pool of plant-community-specific *nosZ* OTUs, consistent with its higher richness estimates.

### 3.3 Community differentiation and taxonomic reorganization of *nosZ*-type denitrifiers

Ordination analyses revealed clear differentiation in *nosZ*-type denitrifier community composition among the four dominant plant communities. In the PCA, PC1 and PC2 accounted for 23.84 and 13.15% of the total variation, respectively, together explaining 36.99% of community variation (Figure 2a). Samples from different plant communities occupied distinct regions of the ordination space, although some overlap remained among groups.

**Figure 2:**
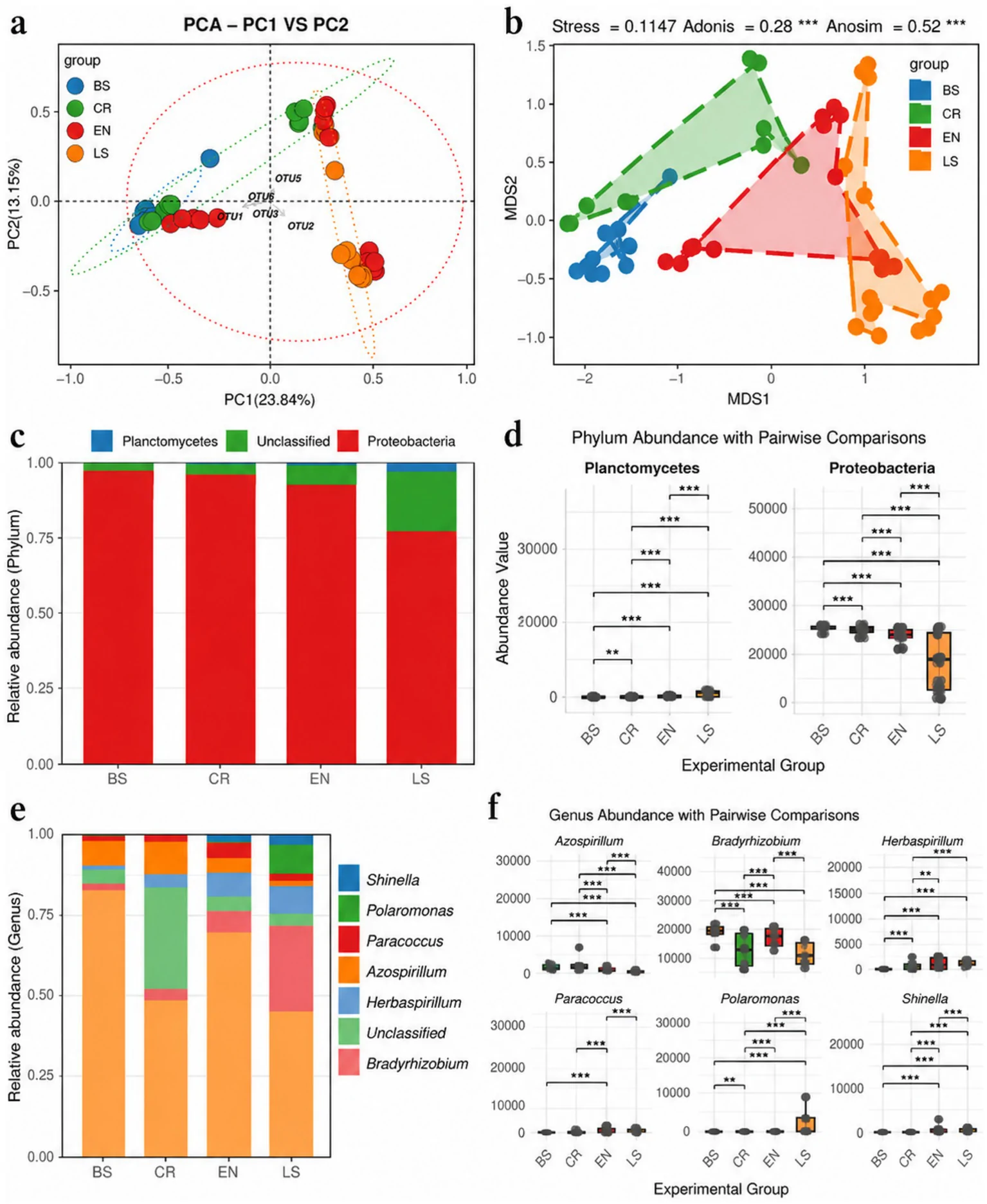
Differentiation and taxonomic composition of *nosZ*-type denitrifier communities across dominant plant communities. **(a)** Principal component analysis (PCA) of *nosZ*-type denitrifier communities across the four dominant plant communities. PC1 and PC2 explained 23.84 and 13.15% of the total variation, respectively. Arrows labeled OTU1–OTU6 represent the six OTUs with the strongest contributions to the PCA ordination. The direction of each arrow indicates its association with the ordination axes, whereas arrow length reflects the relative magnitude of its loading on PC1 and PC2. **(b)** Non-metric multidimensional scaling (NMDS) ordination showing differences in community composition among BS, CR, EN, and LS. The NMDS solution had a stress value of 0.1147, and significant among-group differences were supported by Adonis (*R*^2^ = 0.28, *p* < 0*.001*) and ANOSIM (*R* = 0.52, *p* < 0.001). **(c)** Relative abundance of major phyla within the *nosZ*-type denitrifier community. **(d)** Pairwise comparisons of sequencing-derived abundances of the major phyla, including Planctomycetes and Proteobacteria. **(e)** Relative abundance of dominant genera within the *nosZ*-type denitrifier community. **(f)** Pairwise comparisons of sequencing-derived abundances of six dominant genera, including *Azospirillum, Bradyrhizobium, Herbaspirillum, Paracoccus, Polaromonas, an*d *Shinella. BS*, *Blysmus sinocompressus; CR*, *Carex regescens; EN*, *Elymus nutans; LS*, *Leymus secalinus. As*terisks indicate significant pairwise differences (ns indicates no significant difference, *p < 0.05, **p < 0.01, ***p < 0.001, and ****p < 0.0001). Abundance values in panels **(d)** and **(f)** are sequencing-derived abundance measures and should not be interpreted as absolute microbial abundance.

The differentiation was more evident in the NMDS ordination (Figure 2b). The NMDS configuration showed an acceptable fit (Stress = 0.1147), and multivariate tests confirmed significant differences in community composition among the four plant-community types (Adonis *R*^2^ = 0.28, *p* < 0.001; ANOSIM *R* = 0.52, *p* < 0.001). BS and LS samples were particularly separated in ordination space, while CR and EN occupied additional, partly distinct regions, indicating pronounced plant-community-associated differentiation in *nosZ* community composition.

At the phylum level, Proteobacteria dominated the *nosZ*-type denitrifier communities in all four plant-community types (Figure 2c). Its relative contribution was particularly high in BS and CR but decreased in EN and, more markedly, in LS. In contrast, the proportion of unclassified taxa increased toward LS, while Planctomycetes represented only a minor fraction of the overall community. Pairwise comparisons of sequencing-derived abundance values further identified significant differences in both Proteobacteria and Planctomycetes among several plant-community pairs (Figure 2d).

At the genus level, *Bradyrhizobium* represented the predominant component of the *nosZ*-type community, although its relative contribution varied considerably among plant communities (Figure 2e). *Mesorhizobium*, *Herbaspirillum*, *Azospirillum*, *Paracoccus*, *Polaromonas*, *Shinella*, and unclassified taxa also contributed appreciably to community composition, with their relative representation differing among BS, CR, EN, and LS. Consistent with these compositional patterns, significant between-group differences in sequencing-derived abundance were detected for *Azospirillum*, *Bradyrhizobium*, *Herbaspirillum*, *Paracoccus*, *Polaromonas*, and *Shinella* (Figure 2f). Together, these results indicated that the four dominant plant communities differed not only in overall *nosZ* community composition but also in the relative representation of several dominant taxonomic groups.

### 3.4 Environmental associations of *nosZ*-type denitrifier diversity and community structure

The relative importance of environmental variables differed substantially between *nosZ* community diversity and taxonomic structure. For community diversity, belowground biomass showed the largest contribution (21.1%), followed by aboveground biomass (9.7%), soil temperature (9.2%), TC (8.7%), and TN (7.4%) (Figure 3a). Ammonium nitrogen, CNR, soil moisture, pH, and nitrate nitrogen showed comparatively smaller contributions.

**Figure 3:**
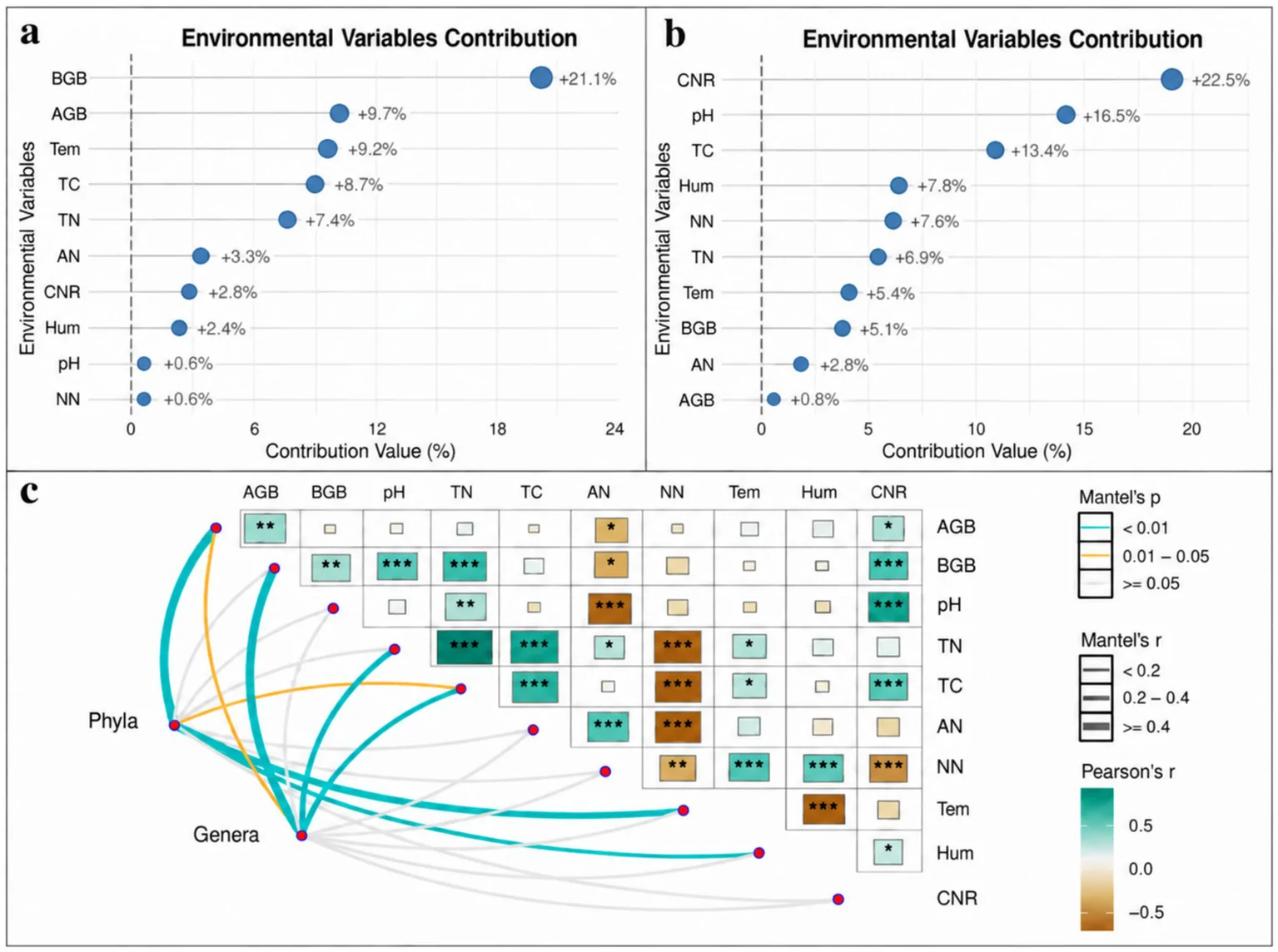
Environmental associations with the diversity and taxonomic structure of *nosZ*-type denitrifier communities. **(a)** Relative contributions of vegetation and soil environmental variables to variation in *nosZ*-type denitrifier community diversity. **(b)** Relative contributions of environmental variables to variation in *nosZ* community taxonomic structure. Values adjacent to the points indicate the contribution of each environmental variable (%). **(c)** Mantel relationships between *nosZ* community composition at the phylum and genus levels and vegetation–soil environmental variables, together with Pearson correlations among environmental variables. In the Mantel network, line color denotes the Mantel test *p*-value category (*p* < 0.01, 0.01 ≤ *p* < 0.05, or *p* ≥ 0.05), whereas line width represents the magnitude of Mantel’s *r*. In the correlation matrix, square color indicates the direction of Pearson’s *r*, with green and brown representing positive and negative correlations, respectively, whereas square size represents the magnitude of Pearson’s *r*. Asterisks denote the significance of Pearson correlations (*p < 0.05, **p < 0.01, and ***p < 0.001), whereas cells without asterisks indicate nonsignificant correlations (*p* ≥ 0.05). AGB, aboveground biomass; BGB, belowground biomass; Tem, soil temperature; Hum, soil moisture; TC, total carbon; TN, total nitrogen; AN, ammonium nitrogen; NN, nitrate nitrogen; CNR, soil C:N ratio; pH, soil pH.

In contrast, variation in community structure was most strongly associated with soil CNR, which accounted for 22.5% of the total contribution, followed by pH (16.5%) and TC (13.4%) (Figure 3b). Soil moisture (7.8%), nitrate nitrogen (7.6%), TN (6.9%), soil temperature (5.4%), and belowground biomass (5.1%) showed intermediate contributions, whereas ammonium nitrogen and aboveground biomass contributed relatively little. These contrasting rankings indicated that variation in *nosZ* diversity and community structure was associated with different dimensions of the vegetation–soil environment.

Mantel analyses further identified significant relationships between taxonomic community variation and multiple environmental variables (Figure 3c). Both phylum- and genus-level community matrices were associated with subsets of vegetation and soil properties, although the strength and significance of these relationships differed between the two taxonomic levels. The accompanying Pearson correlation matrix also revealed pronounced covariation among environmental variables, particularly among vegetation biomass, soil C and N properties, temperature, moisture, and CNR. Overall, these results showed that the environmental correlates of *nosZ* community diversity were not identical to those associated with taxonomic compositional variation.

### 3.5 Community assembly of *nosZ*-type denitrifiers differed among dominant plant communities

The *β*-nearest taxon index (*β*NTI) was consistently positive across all four dominant plant communities and substantially exceeded the conventional +2 threshold (Figure 4a). *β*NTI did not differ significantly between BS and CR, whereas several other pairwise comparisons were significant. In particular, EN and LS generally exhibited higher *β*NTI values than BS and CR, and LS also showed a significantly higher (*p* < 0.05) distribution than EN. These patterns indicated strong departures of observed phylogenetic turnover from null expectations across all four plant-community types.

**Figure 4:**
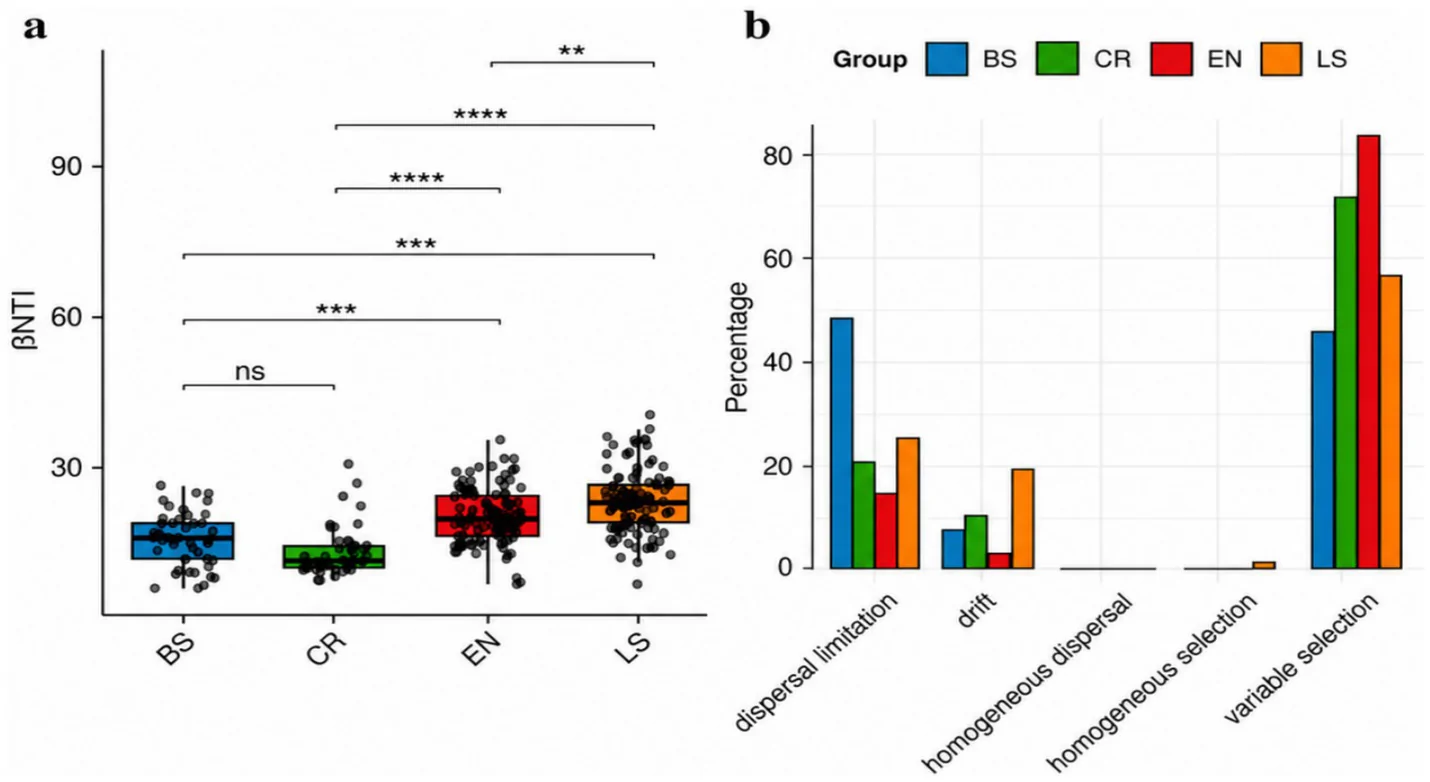
Community assembly processes of *nosZ*-type denitrifiers across dominant plant communities. **(a)** Distribution of the *β*-nearest taxon index (*β*NTI) for *nosZ*-type denitrifier communities across the four dominant plant communities. Boxplots show the median and interquartile range, with individual observations overlaid as points. Pairwise differences among plant-community types are indicated above the boxes. **(b)** Relative contributions of five inferred ecological processes to *nosZ* community assembly, including dispersal limitation, drift, homogeneous dispersal, homogeneous selection, and variable selection. Individual points represent pairwise *β*NTI estimates of phylogenetic turnover between samples and therefore should not be interpreted as independent biological replicates. BS, *Blysmus sinocompressus*; CR, *Carex regescens*; EN, *Elymus nutans*; LS, *Leymus secalinus* (NS indicates no significant difference, *p < 0.05, **p < 0.01, ***p < 0.001, and ****p < 0.0001).

Partitioning of inferred ecological assembly processes revealed substantial differences in their relative contributions among plant communities (Figure 4b). Variable selection represented a major component of *nosZ* community assembly in all four groups and was particularly prominent in CR and EN. In BS, variable selection and dispersal limitation made comparatively similar contributions, whereas dispersal limitation accounted for a smaller fraction in CR, EN, and LS. The process classified as drift contributed relatively little in BS, CR, and EN but represented a larger fraction in LS. Homogeneous selection and homogeneous dispersal contributed only minimally across all four plant-community types. Overall, the inferred assembly patterns were characterized by a strong contribution of variable selection, while the relative balance between selection, dispersal-related processes, and drift differed among dominant plant communities.

## 4 Discussion

### 4.1 Dominant plant communities were associated with distinct *nosZ* diversity and taxonomic composition

The pronounced differences in *nosZ*-type denitrifier diversity and composition among the four dominant plant communities indicate that spatial variation in vegetation was closely accompanied by differentiation of the N₂O-reducing microbial community. In particular, LS supported the highest ACE and Chao1 richness and the largest pool of unique OTUs, whereas the ordination analyses showed clear compositional separation among BS, CR, EN, and LS. Such patterns are consistent with the broader view that plant communities can generate distinct belowground ecological templates through differences in root biomass, rhizodeposition, litter inputs, nutrient acquisition, and modification of soil physicochemical conditions, thereby selecting different microbial assemblages (Berg and Smalla, 2009; Le Roux et al., 2013; Wu et al., 2017). Plant-associated differentiation has also been demonstrated specifically for denitrifying microorganisms: *nosZ* communities differed between meadow and forest soils and were linked to vegetation type and soil C:N conditions, while rhizosphere *nosZ* assemblages in wetlands varied with plant species and associated environmental conditions (Rich et al., 2003; Ruiz-Rueda et al., 2009). At a broader scale, wetland vegetation effects on denitrification vary among communities dominated by different plant species, further indicating that plant identity and the environmental conditions accompanying plant dominance can influence microbial N transformations (Alldred and Baines, 2016). More recent work has shown that the establishment of root-associated N₂O-reducing communities depends jointly on plant and soil filters, rather than on plant identity alone (Graf et al., 2022), and precipitation-induced environmental changes in the Qinghai Lake alpine wetland were likewise accompanied by substantial reorganization of *nosZ*-type communities (Zhang et al., 2025). The dominance of Proteobacteria and particularly *Bradyrhizobium* in our dataset is also consistent with earlier *nosZ*-based surveys in which members of the Rhizobiaceae and other Proteobacteria represented major components of N₂O-reducing communities (Rich et al., 2003). Collectively, these results suggest that the four dominant plant communities represent distinct vegetation–soil habitat contexts for *nosZ*-harboring microorganisms; however, because vegetation and soil properties covary in this observational study, the observed community differentiation should be interpreted as plant-community-associated rather than as a direct causal effect of plant identity.

### 4.2 Diversity and community composition were associated with different environmental dimensions

A particularly notable feature of our results was the clear divergence between the environmental variables associated with *nosZ* community diversity and those associated with taxonomic composition. Belowground biomass made the largest contribution to variation in diversity, followed by aboveground biomass, soil temperature, TC, and TN, whereas compositional variation was associated more strongly with soil CNR, pH, and TC. Such decoupling is ecologically plausible because different attributes of denitrifier communities need not respond synchronously to environmental variation. Across contrasting riparian and agricultural soils, for example, denitrifier abundance, community structure, gene expression, and activity showed distinct responses, indicating that different components of the denitrifier community can be regulated by different environmental constraints (Dandie et al., 2011). At broader spatial scales, soil physicochemical properties, particularly pH, explain substantial variation in the distribution of N-cycling microbial groups, demonstrating that edaphic filtering can operate independently of land-use or spatial effects (Bru et al., 2011). The comparatively strong association of diversity with belowground biomass in our study may therefore reflect the integrative influence of the rhizosphere rather than a direct effect of root biomass itself. Roots simultaneously modify carbon availability, oxygen status, nutrient supply, and microsite conditions, and experimental manipulation of rhizosphere N availability and waterlogging has shown that these changes can alter *nosZ*-bearing community structure and denitrifier activity, with root-derived C providing an important mechanistic link (Hamonts et al., 2013). Temperature and moisture can additionally influence microbial activity and substrate availability without producing equivalent changes in *nosZ* community structure, further emphasizing that environmental sensitivity depends on the community attribute being considered (Stres et al., 2008). In contrast, the prominence of CNR, pH, and TC for taxonomic composition suggests stronger niche-based sorting of community membership along soil chemical gradients. Soil pH can influence denitrification at multiple biological levels, including gene expression, enzyme maturation, and the kinetics of N₂O reduction (Liu et al., 2010), and field experiments have shown that *nosZ* community composition can respond significantly to pH even when other N-cycling groups respond to different environmental variables (Zhang et al., 2017). Consistent with this interpretation, land-use-associated changes in soil properties were accompanied by substantial shifts in *nosZ* diversity and composition on the Loess Plateau (Xu et al., 2019), while recent metagenomic evidence demonstrates that contrasting pH conditions can select markedly different N₂O-reducing microbiomes (Sun et al., 2024). Taken together, these observations support the interpretation that the maintenance of *nosZ* diversity and the sorting of taxonomic composition are associated with partly different dimensions of the vegetation–soil environment. Nevertheless, the strong covariance among environmental variables in our dataset means that the contribution values should be interpreted as measures of statistical importance rather than independent causal effects; accordingly, BB, CNR, pH, and TC are best regarded as candidate environmental filters associated with *nosZ* community organization rather than variables with experimentally demonstrated causal effects.

### 4.3 Deterministic environmental selection dominated *nosZ* community assembly

The consistently positive *β*NTI values, all substantially exceeding +2, indicate that phylogenetic turnover of the *nosZ*-type denitrifier community deviated strongly from null expectations and, within the null-model framework applied here, was predominantly associated with variable selection. This result moves beyond the compositional differences described by PCA and NMDS by suggesting that community differentiation among plant-associated habitats was not simply the outcome of unconstrained stochastic turnover. Microbial communities are generally assembled through the simultaneous action of selection, dispersal, diversification, and demographic stochasticity, and the relative importance of these processes depends strongly on environmental context and spatial scale (Nemergut et al., 2013). Environmental heterogeneity can strengthen deterministic sorting because spatially contrasting habitats impose different fitness constraints on microbial lineages, although the magnitude of this effect varies substantially among ecologically distinct microbial groups (Powell et al., 2015). Soil pH provides a particularly well-established example: increasingly restrictive pH conditions can shift bacterial assembly toward stronger deterministic selection, whereas environments imposing weaker filters can permit a larger contribution from stochastic processes (Tripathi et al., 2018). Against this conceptual background, the predominance of variable selection in CR and EN is consistent with strong ecological sorting of *nosZ* lineages among heterogeneous vegetation–soil habitats, while the comparatively greater contribution of dispersal limitation in BS suggests that spatial movement and colonization constraints may also contribute to local community differentiation. The latter interpretation is important because dispersal does not merely homogenize microbial communities: theoretical and experimental studies show that dispersal rate can interact with selection and demographic stochasticity to alter both the reproducibility of community assembly and the resulting community composition (Evans et al., 2017), and direct field manipulation has demonstrated that even relatively low microbial dispersal can measurably affect soil-surface microbiome composition and functioning (Walters et al., 2022). Similarly, large-scale soil surveys indicate that species sorting and dispersal limitation can coexist and that their relative balance contributes to microbial community differentiation across habitats (Jiao et al., 2020). The relatively larger fraction categorized as drift in LS therefore does not contradict the strong overall selection signal; rather, it suggests that local assembly can contain simultaneous deterministic and stochastic components whose relative contributions differ among plant-community contexts. More recent quantitative null-model developments have also emphasized that apparent whole-community assembly patterns can conceal substantial process heterogeneity among individual phylogenetic groups and that categories such as “drift” are statistically inferred residual processes rather than direct observations of ecological events (Ning et al., 2020). Accordingly, within the null-model framework applied here, the *β*NTI and process-partitioning results indicated that *nosZ* community assembly was predominantly associated with variable selection, while the inferred contributions of dispersal-related and stochastic processes differed among BS, CR, EN, and LS.

### 4.4 Ecological implications, study limitations, and future perspectives

The present findings indicate that the four dominant plant-community types surrounding Qinghai Lake were associated with distinct soil *nosZ*-type denitrifier assemblages. Differences in plant biomass, root-derived inputs, litter characteristics, and vegetation-mediated soil microenvironments can generate heterogeneous belowground habitats for soil microorganisms (Berg and Smalla, 2009). Consistent with this interpretation, plant biomass and soil physicochemical properties showed different associations with *nosZ* community diversity and taxonomic composition in the present study. This divergence suggests that community diversity and compositional turnover represent distinct dimensions of microbial organization and may respond differently to vegetation–soil environmental heterogeneity. Similar plant- and soil-associated differentiation has been reported for root-associated *nosZ* communities, supporting the view that plant identity and edaphic context can jointly structure the distribution of this functional guild (Graf et al., 2022). Because the four plant-community types occurred at different sampling sites, however, these patterns should be interpreted as plant-community-associated differentiation rather than as direct effects attributable solely to plant identity.

The null-model results provide an additional ecological perspective on this community differentiation. Variation in the inferred balance among variable selection, dispersal limitation, and undominated stochastic processes suggests that community turnover may reflect both environmental sorting and spatial constraints. Because these processes were inferred from deviations of observed phylogenetic and taxonomic turnover from null expectations rather than measured directly (Nemergut et al., 2013), the relatively high contribution assigned to variable selection is most appropriately interpreted as being consistent with heterogeneous environmental filtering. The observed associations of community composition with soil pH, moisture, temperature, and carbon and nitrogen conditions support this interpretation, although covariance among environmental variables and unresolved spatial dependence preclude attribution to any single environmental factor. Taken together, taxonomic differentiation, environmental associations, and null-model-based assembly inference provide complementary evidence that soil *nosZ*-type denitrifier communities vary systematically across the vegetation–soil mosaic of the Qinghai Lake alpine wetland. These results therefore highlight the value of integrating community composition with assembly-based analyses when examining the spatial organization of functional microbial guilds across naturally occurring plant-community habitats.

Several limitations should nevertheless be considered when interpreting these results. First, sampling was conducted during a single growing-season period, and multiple quadrats were nested within a limited number of spatially distinct sampling sites. Consequently, seasonal dynamics and site-level spatial dependence cannot be fully resolved from the present dataset. Stronger inference regarding plant-community effects will require greater site-level replication and repeated temporal sampling. Second, the *nosZ*-F/1622R primer pair used here represents a conventional *nosZ* amplification strategy. Previous primer evaluations have shown that conventional Proteobacteria-oriented *nosZ* primers may underrepresent *nosZ* diversity in non-Proteobacterial groups, including Firmicutes and Bacteroidetes (Jung et al., 2013). Accordingly, the pronounced dominance of Proteobacteria observed here should be interpreted as the taxonomic structure of the primer-defined fraction of the *nosZ* community rather than as a complete inventory of all N₂O-reducing microorganisms.

Third, DNA-based amplicon sequencing provides information on the occurrence and relative representation of targeted *nosZ* lineages but does not quantify absolute gene abundance, transcription, enzyme activity, or realized N₂O → N₂ fluxes. Likewise, the ecological-process categories inferred from *β*NTI and related null-model analyses are statistical representations of assembly processes rather than direct observations of selection, dispersal, or drift. Future studies should therefore combine broader or clade-resolved *nosZ* assays with qPCR or digital PCR, transcript-level measurements, direct measurements of N₂O and N₂ fluxes, repeated seasonal sampling, and greater site-level replication. Such integration would help distinguish plant-community effects from spatial and temporal variation and allow the community differentiation and assembly patterns identified here to be linked more directly to the ecological functioning of *nosZ*-harboring microorganisms in alpine wetland soils.

## 5 Conclusion

Different dominant plant communities in the Qinghai Lake alpine wetland were associated with pronounced differentiation in the diversity, taxonomic composition, and assembly of *nosZ*-type denitrifier communities. Soils associated with *Leymus secalinus* supported relatively high *nosZ* richness and a large pool of unique OTUs, whereas Proteobacteria and *Bradyrhizobium* dominated the *nosZ*-bearing communities across plant-community types. The diversity and taxonomic composition of *nosZ*-type denitrifiers were associated with partly distinct environmental dimensions, indicating that changes in community diversity and compositional sorting may respond differently to vegetation–soil environmental heterogeneity. Null-model analysis further indicated that community assembly was predominantly associated with variable selection, while the inferred contributions of dispersal limitation and other stochastic processes differed among dominant plant-community habitats. Together, these findings highlight the importance of integrating vegetation–soil heterogeneity, taxonomic differentiation, and community assembly to understand *nosZ*-bearing denitrifier ecology in alpine wetlands, although the consequences for actual N₂O reduction require further validation using absolute abundance, gene-expression, and gas-flux measurements.

## Data Availability

The data analyzed in this study are available in the NCBI repository (https://www.ncbi.nlm.nih.gov) under BioProject accession number PRJNA1515140.
